# Incidental radiological diagnosis of asymptomatic Abernethy malformations—two case reports

**DOI:** 10.1259/bjrcr.20150496

**Published:** 2016-07-29

**Authors:** Ali Shah, Abdul Aziz, Amir Awwad, Greg Ramjas, Yutaro Higashi

**Affiliations:** ^1^Trauma and Orthopaedics, Queen’s Medical Centre, Nottingham University Hospitals, Nottingham, UK; ^2^Sir Peter Mansfield Imaging Centre (SPMIC), Queen’s Medical Centre, Nottingham University Hospitals, Nottingham, UK; ^3^Radiology Department, Queen’s Medical Centre, Nottingham University Hospitals, Nottingham, UK; ^4^Interventional Radiology, Queen’s Medical Centre, Nottingham University Hospitals, Nottingham, UK

## Abstract

The diagnosis of the rare congenital extrahepatic portosystemic shunts is of clinical significance because of the risk of hepatic encephalopathy; liver dysfunction; and associated cardiac, gastrointestinal, vascular, skeletal and genitourinary anomalies. This article describes two varying cases showing the same type of the extrahepatic congenital shunts (Type II). Both the patients were clinically asymptomatic. The first patient initially presented with unprovoked deep venous thrombosis and a staging CT scan was performed to identify any potential underlying malignancy. The second was a polytrauma patient in whom a congenital extrahepatic portosystemic shunt was identified on the CT scan performed to investigate the trauma-related injuries. The first case underwent hepatological investigations, including a fibroscan to rule out liver fibrosis, and was diagnosed as having a Type II congenital malformation, while the second case is under observation post recovery from his traumatic injuries and will be subsequently referred to the hepatology team in the future. Although uncommon, extrahepatic portosystemic shunts can cause significant morbidity and mortality, and all new cases diagnosed radiologically should be further investigated by referring them to a hepatologist.

## Summary

The diagnosis of congenital extrahepatic portosystemic shunts (CEPS) is of clinical significance because of the risk of hepatic encephalopathy; liver dysfunction; and associated cardiac, gastrointestinal, vascular, skeletal and genitourinary anomalies. While the actual incidence and clinical importance of CEPS in asymptomatic patients is not known, they are extremely rare in healthy individuals.

### Symptomatology and investigations

This article describes two varying cases showing the same type of extrahepatic congenital shunts (Type II). Both the patients were healthy and clinically asymptomatic. The first patient initially presented with unprovoked deep venous thrombosis (DVT) and a staging CT scan was performed to identify any potential underlying malignancy. The second was a polytrauma patient in whom CEPS was identified on the CT scan performed to evaluate the traumas.

The first case underwent hepatological investigations, including a fibroscan to rule out liver fibrosis, and was diagnosed as having a Type 1 congenital malformation, while the second case is under observation post recovery from his traumatic injuries and will be subsequently referred to the hepatology team in the future.

### Conclusions

Although uncommon, extrahepatic portosystemic shunts can cause significant morbidity and mortality and all new cases diagnosed radiologically should be further investigated by referring them to a hepatologist.

## Case report 1

### Clinical presentation

A 68-year-old female was referred to the haematology clinic with left-sided above-knee DVT, which was essentially unprovoked as per the obtained clinical history. Her medical history included osteoporosis, osteoarthritis and sciatica. She had undergone a subtotal colectomy with ileorectal anastomosis for large bowel obstruction due to a histologically proven benign stricture secondary to colonic diverticular disease 16 years ago.

Although she was a non-smoker, she had been consuming 20–30 units of alcohol per week for the past many years until 4 years ago, when her daily intake increased by an additional 10 units. She often indulged in binge drinking, mainly for the pain associated with her musculoskeletal and rheumatological comorbidities. There was no clinical history to suggest any liver abnormality (*e.g.* jaundice, pedal oedema, ascites, encephalopathy, gastrointestinal bleeding) or symptoms suggestive of cardiac overload such as shortness of breath or history of cardiac ischaemic disease.

### Investigations

Haematological and liver function tests (LFTs) revealed asymptomatic mild thrombocytopenia that had been ongoing since 2006. Mild derangement of the LFTs was also noted (Table 1).

A portovenous phase CT scan was performed by the haematologist to look for any possible underlying malignancy as the cause of the DVT. The scan showed bulky enlargement of the left thyroid lobe with multiple nodules. There was neither any supraclavicular, thoracic or axillary lymphadenopathy nor any focal lung lesions. The gallbladder, pancreas, spleen, adrenal glands and kidneys were also unremarkable.

**Figure 1.**
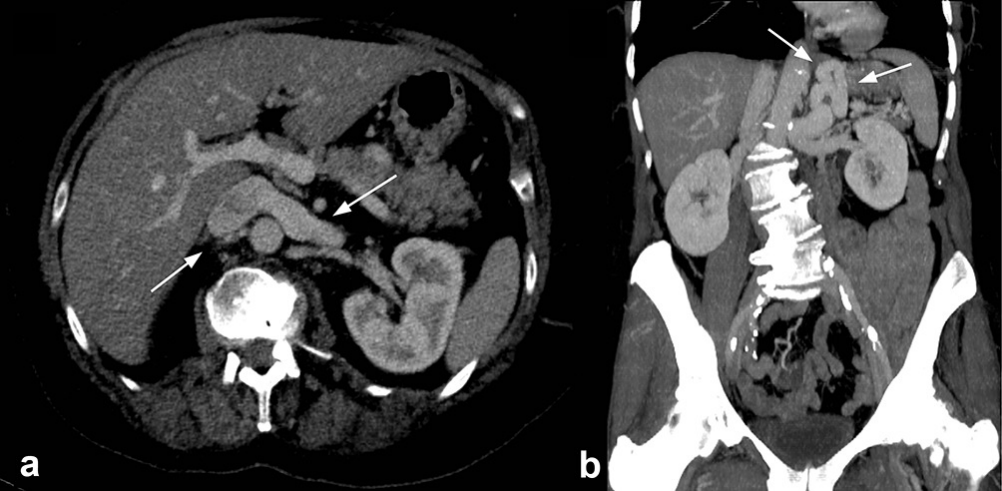
(a) Axial CT image (1 mm, maximum intensity projection) showing an engorged left renal vein posteriorly, relative to the portal vein, due to the congenital extrahepatic portosystemic shunt in a 68-year-old asymptomatic female patient. (b) Coronal reconstructed CT image (5 mm, maximum intensity projection) showing a tortuous and dilated left gastric (shunt) and splenic vein within the left upper abdomen and communicating superior mesenteric vein and portal vein with the left renal vein and inferior vena cava (Supplementary Video 1a,b).

However, there was an incidental finding of an extrahepatic portosystemic connection, with an enlarged vein arising from the portal vein just superior to the confluence of the superior mesenteric and splenic veins. This was seen to anastomose with an engorged left adrenal vein and ultimately drain into the left renal vein. The hepatic portal vein was evidently patent. The appearance of the liver was consistent with fatty infiltration but was otherwise unremarkable (Figure 1a,b).

### Diagnosis and management

The radiologist reporting the CT scan recommended a hepatology clinic referral, which was subsequently organized. During the clinic visit, a full assessment, including physical examination, was conducted. Contrary to any plausible clinical expectations, no central or peripheral signs of cardiac or chronic hepatic disease were identified. There were neither audible murmurs on auscultation nor any evidence of hyperdynamic circulation. The liver was not palpable, and there was no free fluid in the abdomen. There was, however, a mildly enlarged spleen, about 2 cm below the left costal margin; this was also confirmed on the CT images.

The patient, however, was still asymptomatic with a Type II Abernethy malformation, and her previous surgical history did not seem to qualify as an iatrogenic cause for this shunt. Therefore, further investigations were conducted. These included a non-invasive liver screen with immunoglobulins, autoimmune and viral hepatitis (hepatitis B and C) markers; a fibroscan with a view to proceed to liver biopsy; and an endoscopy to look for portal hypertension and varices.

On her subsequent review by the hepatology team, the fibroscan showed a median liver stiffness measurement value of 5.5 kPa (normal healthy adult < 7.0 kPa, median 5.3 kPa), which was within the normal range to exclude liver fibrosis. Additionally, a subsequent non-invasive liver screen was also negative. The platelet count on the most recent pathology test was normal (platelet count 154). Given these results, chronic liver disease and portal hypertension were deemed unlikely and the most likely cause of her shunt was believed to be a long-standing congenital anomaly, hence a liver biopsy was not indicated.

### Outcome

Given the above findings in the absence of any local and/or systemic complications, she was discharged back to the care of her general practitioner without any further follow-up planned.

## Case report 2

### Clinical presentation

A 56-year-old male was brought in by the regional ambulance team to our trauma centre (level 1) after being involved in a high speed road traffic accident. His past medical history included schizophrenia. Otherwise, he was fairly fit and well, with no significant comorbidities.

He was seen and assessed by the trauma team and had a series of investigations and imaging studies, which included performing a CT scan to evaluate the traumas. He was found to have acute multiple traumatic injuries, all right-sided, with several fractures of the right upper and lower limbs, and the right hemipelvis.

### Investigations

**Figure 2.**
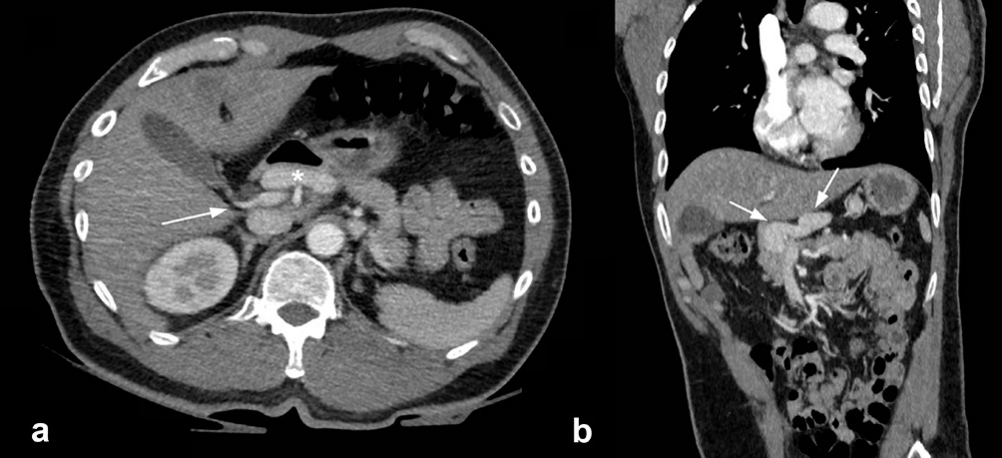
(a) Axial CT image of the upper abdomen (1 mm maximum intensity projection) showing a distended left gastric vein acting as a shunt (asterisk) anterior to the portal vein (arrow), which is seen anterior to the normal size inferior vena cava. (b) Coronal reconstructed CT image (1 mm, maximum intensity projection) showing the shunt joining at the confluence of the superior mesenteric and splenic veins (side-to-side anastomosis) within the upper abdomen, surrounding the head of the pancreas (Supplementary Video 2a,b).

The CT scan revealed an abnormal enhancing and distended extrahepatic portosystemic communication between the left renal vein/inferior vena cava and the splenic vein. A markedly hypoplastic portovenous system was also noted, likely due to considerable flow diversion into the systemic veins without any intrahepatic shunts identified. The findings suggested an incidental Type II CEPS, draining into the left renal vein (Figure 2a,b).

His LFTs on admission were normal, as detailed in Table 1. Following his management, he spent a significant amount of time in high dependency care and was repatriated to his base hospital for further management.

**Table 1.** Patients’ test results (haematology and biochemistry)

Tests	Case 1	Case 2	Normal range
Haemoglobin	140	100	115–165 g l^–1^
Platelets	142	117	150–450 × 10^8^ l^–1^
Bilirubin	24	25	0–21 μmol l^–1^
Alkaline phosphatase	60	70	40–130 U l^–1^
Alanine aminotransferase	32	34	0–45 U l^–1^
Aspartate aminotransferase	51	74	0–35 U l^–1^

### Outcome

There were no findings on history taking or physical examination to suggest any relevant symptomatology, and thus a full hepatological screen (viral screen, immunoglobulins, neutrophil cytoplasmic antibody level and antinuclear antibody level) was not carried out owing to the circumstances.

## Discussion

This case series has limitations, as the second patient is still recovering from injuries caused by the trauma and therefore has not had a formal hepatology review.

There are two types of congenital portosystemic shunts, intrahepatic and extrahepatic. Intrahepatic shunts include persistent patent ductus venosus and congenital hepatic vascular lesions, where the branches of the portal vein, after its division, join the hepatic veins or the inferior vena cava directly. In extrahepatic shunts, a systemic vein joins the portomesenteric vasculature before the division of the portal vein. Our case series only deals with extrahepatic portosystemic shunts.

CEPS were first described by John Abernethy in 1793. They are extremely uncommon, especially in an asymptomatic individual. A systematic review spanning over 29 years (1982–2011) of publications yielded a total of 80 studies with only 49 cases of CEPS.^1^ Two patients had no symptoms at the time of diagnosis.^1^ Therefore, while the exact incidence of CEPS is difficult to quantify, they are a rarity.^1^ They are, however, of significance because of their associated anomalies involving the cardiac and the hepatic systems along with the risk of hepatic encephalopathy.^2^ These are detailed in Table 2.^3^ Furthermore, shunts may affect the hepatic immune surveillance owing to the toxins and pathogens bypassing the liver. They may also influence the systemic concentrations of compounds metabolized by the liver and potentially become a risk factor in patients with gastrointestinal tumours for earlier pulmonary metastasis.^1^

**Table 2.** Associated anomalies

Circulatory	Gastrointestinal	Genitourinary
• Atrial septal defect • Congenital aortic valve stenosis • Dextrocardia • Mesocardia • Patent ductus arteriosus • Patent foramen ovale • Tetralogy of Fallot • Ventricular septal defect • Interruption of the inferior vena cava • Double inferior vena cava	• Biliary atresia • Choledochal cyst • Intrahepatic gallbladder • Polysplenia	• Bilateral ureteropelvic stenosis • Crossed fused renal ectopia • Hypospadias • Multicystic dysplastic kidney • Vesicoureteral reflux

There are two types of CEPS. Type I, in which there is absence of intrahepatic portal venous supply, is due to the complete diversion of portal blood into systemic circulation (end-to-side shunt).^3^ It is more common in females. ^2^ In Type II, the intrahepatic supply *via* the portal vein is preserved but some of the portal flow is diverted into a systemic vein (side-to-side shunt). The age of diagnosis varies from 31 weeks of intrauterine life to 76 years.^2^

Portosystemic shunts arising without concomitant liver disease or portal hypertension^4^ can also occur owing to abdominal trauma, prior surgery or postnatal massive necrosis secondary to either viral or hepatotoxic injury.^5^ Most extrahepatic systemic shunts involve a mesenteric vein and the vena cava.^5^

There are different modalities for the diagnosis of CEPS. Ultrasound scan is the investigation of choice in symptomatic infants owing to its non-invasive nature; it requires no sedation and does not expose the patient to radiation. Often CEPS is an incidental finding when an ultrasound scan is performed for another purpose. Doppler ultrasound scan can detect the flow direction. The limitations of ultrasound scan include failure to fully visualize all associated shunts.^6^ A recently published systematic review showed that 31 out of 112 patients (27.6%) required two or more different modalities for diagnosis.^1^

For this purpose, CT and MR angiography scans are recommended. Radiological Society of North America recommends MR angiography to be considered first owing to its reliability in assessing hepatic vascular anatomy without exposing the patient to radiation.^3^ With the development of multidetector CT scanners, it has been reported that its spatial resolution in the detection of small vascular branches is superior to that of MR angiography.^7^ Currently, Doppler and CT angiography are the most common radiological techniques used for making a diagnosis.^1^

Conventional angiography, on the other hand, has risks of radiation, vascular injury and anaesthesia, and is not routinely required owing to MRI or CT scan findings leading to a diagnosis in most cases. Techniques such as indirect mesenteric portovenography can clarify the portal system anatomy, whereas percutaneous transhepatic portography can assist with selective embolization in Type II shunts as well as visualization of features of an extrahepatic shunt.^3^

Other investigations include portal scintigraphy performed with rectal administration of iodine 123 iodoamphetamine, the isotope of which are absorbed by the inferior mesenteric vein and carried to the liver. If a shunt is present, the isotope will be detected on the images of both the liver and lungs, otherwise only on the images of the liver. Thus nuclear studies can calculate shunt ratios in Type II CEPS.^3^

Liver biopsy, although not necessary, may help to differentiate between Type I and II CEPS by showing small portal venules within the portal triads in Type I, a finding that cannot be seen with imaging tests, and hence can influence the type of management.

Treatment of CEPS is dependent on patient factors and presentation. Children are usually asymptomatic owing to their central nervous system being less sensitive to the effects of possible hyperammonaemia. Therefore, they may need to be closely monitored, as medical therapy and dietary changes, for example, a low protein diet, can be used to manage most mild metabolic abnormalities.^3^

For symptomatic patients, it is prudent to determine the type of shunt. While Type II shunts can be occluded either surgically or by embolization (percutaneous transcatheter coil placement), Type I shunts are the only drainage route for splenic and mesenteric blood, and hence liver transplantation is the choice of treatment in symptomatic patients.^3^

## Learning points

1. CEPS represent a rare but important condition with many associated anomalies.
2. Identifying CEPS on scans early can assist with prompt diagnosis and management.
3. The diagnosis is made radiologically and the approach is multidisciplinary, with the radiologist, hepatologist and specialist for each of the systems involved.

## Consent

Appropriate consent was obtained to publish the report from the first patient; however, informed consent could not be obtained from the second patient or the next of kin despite exhaustive efforts. Both cases have been sufficiently anonymized to protect patient identity.
